# Age- and deprivation-related inequalities in identification of people at high risk of type 2 diabetes in England

**DOI:** 10.1186/s12889-024-19571-x

**Published:** 2024-08-10

**Authors:** Ruth Watkinson, Emma McManus, Rachel Meacock, Matt Sutton

**Affiliations:** Health Organisation, Policy and Economics, Division of Population Health, Health Services Research and Primary Care, School of Health Sciences, Faculty of Biology, Medicine and Health, The University of Manchester, Williamson Building, Oxford Road, Manchester, M13 9PL UK

**Keywords:** Type 2 Diabetes, Prediabetes, Non-diabetic hyperglycaemia, Inequalities, Intermediate hyperglycaemia

## Abstract

**Background:**

Early detection of intermediate hyperglycaemia, otherwise known as non-diabetic hyperglycaemia (NDH) is crucial to identify people at high risk of developing type 2 diabetes mellitus (T2DM) who could benefit from preventative interventions. Failure to identify NDH may also increase the risks of T2DM-related complications at the time of T2DM diagnosis. We investigate sociodemographic inequalities in identification of NDH in England.

**Methods:**

We used nationwide data from the English National Health Service (NHS) National Diabetes Audit, which includes all people who were newly identified with NDH (*N* = 469,910) or diagnosed with T2DM (*N* = 222,795) between 1st April 2019 and 31st March 2020. We used regression models to explore inequalities in the under identification of NDH by area-level deprivation and age group.

**Results:**

Of those with a new T2DM diagnosis, 67.3% had no previous record of NDH. The odds of no previous NDH being recorded were higher amongst people living in more deprived areas (Odds ratio (OR) 1.15 (95% confidence intervals (CI) [1.12, 1.19]) most deprived (Q1) compared to least deprived (Q5) quintile) and younger individuals (OR 4.02 (95% CI [3.79, 4.27] under 35s compared to age 75–84)). Deprivation-related inequalities persisted after stratification by age group, with the largest inequalities amongst middle and older age groups. People living in more deprived areas and younger people also had shorter recorded NDH duration before progression to T2DM, and higher T2DM severity at the time of diagnosis.

**Conclusions:**

There is under identification of NDH relative to diagnosis of T2DM amongst people living in more deprived areas and particularly amongst younger people, resulting in missed opportunities for targeted T2DM prevention efforts and potentially contributing to inequalities in T2DM prevalence and severity. More active NDH case-finding amongst these groups may be an important first step in helping to reduce inequalities in T2DM.

**Supplementary Information:**

The online version contains supplementary material available at 10.1186/s12889-024-19571-x.

## Background

Early detection of intermediate hyperglycaemia is important for identifying individuals at high risk of developing type 2 diabetes mellitus (T2DM) so that: (1) individuals are informed about their risk of developing T2DM; (2) there are opportunities for interventions to prevent or delay T2DM; and (3) individuals can be monitored to identify progression to T2DM early, minimising the risk of complications linked to uncontrolled T2DM [1].

In the UK there is a focus on identifying people with non-diabetic hyperglycaemia (NDH), otherwise known as intermediate hyperglycaemia or prediabetes [2, 3]. NDH identification in England has increased from under 100,000 records per year in 2010, to over 450,000 in 2019 [4], increasing opportunities for T2DM prevention. Some of this increase coincided with the 2018 national rollout of the National Health Service (NHS) Diabetes Prevention Programme (DPP) [4], which is a behaviour-change intervention to prevent or delay T2DM amongst individuals at high risk, including those identified with NDH [1]. The DPP has been shown to be effective in delaying and potentially preventing T2DM at the population level [5] and DPP referral is associated with reduced conversion from NDH to T2DM [6].

However, multiple steps along the DPP care pathway may widen pre-existing inequalities in the burden of T2DM across different population groups [7]. For example, there are inequalities during the referral process, with poorer quality general practices making fewer referrals (where clinical quality was measured using performance on the Quality and Outcomes Framework (QQF)) [8]. Following referral, DPP uptake, retention, and completion was lower amongst men, younger people, people living in more deprived areas, and people from ethnic minority backgrounds [9]. For the same groups, there was less improvement in intermediate outcomes (weight loss and blood glucose) amongst those who completed the programme [10]. Identifying individuals with NDH in their clinical record is one route to DPP referral, so inequalities in NDH identification may feed forward into further T2DM inequalities.

Diagnosis rates of T2DM and identification rates of NDH increase with age [4]. However, to the best of our knowledge, there is no existing analysis of age-related inequalities in the identification of NDH/intermediate hyperglycaemia prior to T2DM diagnosis. Although fewer in number, younger people at high risk of T2DM are an important subpopulation, as diagnosis rates of T2DM are increasing most rapidly in this group [11]. In addition, people with young-onset T2DM (aged under 40) typically experience more rapid progression from NDH to T2DM, higher severity T2DM at the time of diagnosis, and more T2DM-related complications compared to people who are diagnosed with T2DM later in life [4, 12–15].

NHS electronic health record data suggests there are minimal deprivation-related inequalities in the population identified with NDH: 21% of people with recorded NDH lived in the 20% most deprived areas in 2020/2021 [4]. However, far wider deprivation-related inequalities were identified when NDH prevalence was determined using blood tests in a cohort study of older adults, which suggests NDH may be under-identified in more deprived areas [13]. Therefore, although the distributions of recorded NDH and T2DM prevalence are known, it is unclear whether there are inequalities in the under-identification of NDH.

In this paper, we use a robust national dataset to explore deprivation-related inequalities in the identification of NDH relative to T2DM diagnosis levels. We also investigate how deprivation-related inequalities vary by age group and generate new insights into age-related inequalities in the under-identification of NDH.

## Methods

### Data and populations

We used pseudo-anonymised, routinely collected data from the NDA Core and NDH components of the National Diabetes Audit Core module [16], obtained from NHS Digital (DARS-NIC-196221-K4K3Y). This was part of the DIPLOMA research project which has ethical approval from the NHS Digital Independent Group Advising on the Release of Data and the North West Greater Manchester East Research Ethics Committee (reference 17/NW/0426). The data collected as part of the NDA Core covered individuals diagnosed with T2DM, and as such individuals diagnosed with other types of diabetes, such as type 1 and gestational diabetes, were not included in the NDA Core dataset.

We combined data from three audit periods: the first covered a period of 12 months: 1st April 2017 – 31st March 2018, and the subsequent two covered a period of 15 months: 1st January 2018 – 31st March 2019, and 1st January 2019 – 31st March 2020, retaining just the first record each of NDH and/or T2DM per individual. We do not examine data from beyond March 2020 due to the widespread disruption to routine primary care caused by the COVID-19 pandemic. Individuals are included in the audit based on T2DM, NDH, or other diabetes-related Read codes extracted from primary care electronic health records [16]. We generated two datasets: one containing all individuals identified with NDH, and another for those diagnosed with T2DM. After excluding people with missing date of diagnosis or identification, we restricted the main samples to individuals newly recorded with (1) NDH or (2) a new diagnosis of T2DM between 1st April 2019 and 31st March 2020, and with complete data for age and area-level deprivation (Figure S1). Within the T2DM dataset, we merged in individual-level NDH identification data (recorded during any time period) to allow identification of NDH recording prior to T2DM diagnoses (Figure S1).

We obtained 2019 mid-year population estimates for England at lower-layer super output area (LSOA) level, stratified by 5-year age group and 2019 index of multiple deprivation (IMD) quintile from the Office of National Statistics [17].

### Variables

We grouped age in years as follows: 15–34, 35–44, 45–54, 55–64, 65–74, 75–84, and 85 plus. We assigned IMD quintile based on individual’s LSOA of residence [18].

We derived binary indicators for new 2019/20 T2DM diagnoses and NDH identification based on Read code type and date in the audit. We derived estimates of the 2019/20 population at risk of T2DM for each age group within each deprivation quintile by subtracting all pre-existing (up to 31st March 2019) T2DM cases recorded in the audit data from 2019 mid-year population estimates [17]. To estimate the population at risk of NDH, we subtracted both pre-existing T2DM and NDH cases.

Within the population with T2DM, we used a binary indicator variable for not having previously been identified with NDH at any time prior to T2DM diagnosis. We derived a numeric variable for NDH duration, as the difference in days between T2DM diagnosis and recorded NDH dates (where both exist). We derived binary indicator variables for the presence of recorded HbA1c at the time of NDH identification and (separately) T2DM diagnoses, defining ‘at the time’ as plus or minus three months between the date of diagnosis and HbA1c result. We used HbA1c values measured in mmol/l as an indicator of severity at the time of diagnosis. For reference, NDH is defined as HbA1c of 42–47 mmol/l and T2DM as HbA1c of 48 mmol/l or above [4].

### Statistical analysis

All statistical analysis was done using Stata 14. Where population numbers from National Diabetes Audit data are reported, values are rounded to the nearest 5, as stipulated in the data sharing agreement.

In all analyses, we first estimated inequalities by deprivation using the least deprived quintile (Q5) as the reference group with no additional covariates. We then separately estimated inequalities by age group, using 75–84 as the reference category because this was the group with the highest recorded prevalence of NDH. As populations have substantially younger age structures in more deprived areas (Figure S2), we then estimated deprivation-related inequalities stratified by age group in each case. This stratified approach allows for the complex relationship between population age structures and area deprivation in a fully flexible manner. We used robust standard errors in estimation throughout [19], and present estimates with 95% confidence intervals (CI).

### Models used for each outcome

1. We estimated inequalities in rates of 2019/20 new NDH identification and T2DM diagnosis (separately) per population at risk using Poisson regression, presenting results as incidence rate ratios (IRRs).
2. Within the population who were diagnosed with T2DM during 2019/20, we used logistic regression to estimate inequalities in the outcome of not having a previous NDH diagnosis, presenting results as odds ratios (OR).
3. Amongst individuals with a 2019/20 new T2DM diagnosis and a previous NDH record, we estimated inequalities in recorded NDH duration using Poisson regression. We then computed marginal effects, reporting the contrasts across groups as the estimated difference in duration in days.
4. We used linear regression to estimate inequalities in HbA1c values at the time of NDH identification. Results are presented as the estimated difference in HbA1c (mmol/l) compared to each reference group. HbA1c values at the time of T2DM diagnosis had a skewed distribution, so we first took the natural logarithm of each HbA1c value, then used linear regression. We exponentiated coefficients and multiplied by the constant to obtain estimates of mean difference in HbA1c values (mmol/l) compared to the reference group. In this analysis we added further comparisons, first by whether people were previously identified with NDH, then by whether people were previously identified with NDH stratified by age group. Finally, we looked at whether deprivation and age-related inequalities in severity at the time of T2DM diagnosis differed based on whether people had a previous recording of NDH by re-estimating deprivation and age-related inequalities stratified by previous NDH identification status.

To check potential biases in the presence of data recording HbA1c values, we used logistic regression to estimate inequalities in the odds of having a HbA1c result recorded at the time of NDH identification, including deprivation and age group in the same model, presenting results as ORs. We used the same approach to estimate inequalities in having a HbA1c result at the time of T2DM diagnosis, but also added an indicator for no previous NDH recorded as another predictor.

### Reporting

Results are reported in line with STROBE guidelines [20].

## Results

### Descriptive statistics

Between 1st April 2019 and 31st March 2020 there were 469,910 new cases of NDH, and 222,795 new diagnoses of T2DM recorded in England (Table 1). The mean age at NDH identification was higher (61.0 years) compared to T2DM diagnosis (58.8 years), with a higher proportion of NDH cases amongst the over 75s (19.3%) compared to T2DM diagnoses (14.8%). NDH identification and T2DM diagnosis was more common amongst people living in more deprived areas. However, the magnitude of inequality was greater for T2DM (24.9% in the most deprived quintile (Q1)) than for NDH (21.6% in Q1). Only 32.7% of those diagnosed with T2DM had previously been identified with NDH. Amongst those previously identified with NDH, the median NDH duration was 1,120 days (2.8 years). Median HbA1c at time of NDH identification was 43.0 mmol/l and 50.0 mmol/l for T2DM.

**Table 1:** Descriptive statistics. Demographics and NDH/T2DM-related identification and diagnosis measures for the populations newly identified with NDH or diagnosed with T2DM between 1st April 2019 and 31st March 2020

	**NDH identification**	**T2DM diagnosis**
N (= people with new diagnosis in 2019/20)	469,910	222,795
Age in years, mean (SD)	61.0 (14.8)	58.8 (14.3)
Age group (%)		
15–34	21,650 (4.6)	10,260 (4.6)
35–44	45,280 (9.6)	26,070 (11.7)
45–54	88,665 (18.9)	51,195 (23.0)
55–64	111,400 (23.7)	56,045 (25.2)
65–74	112,110 (23.9)	46,105 (20.7)
75–84	70,395 (15.0)	25,910 (11.6)
85 +	20,415 (4.3)	7,220 (3.2)
IMD quintile (%)		
Q1 (most deprived)	101,580 (21.6)	55,460 (24.9)
Q2	100,415 (21.4)	50,540 (22.7)
Q3	97,775 (20.8)	45,640 (20.5)
Q4	89,010 (18.9)	39,150 (17.6)
Q5 (least deprived)	81,125 (17.3)	32,005 (14.4)
NDH recorded before T2DM diagnosis (any duration) (%)		
No		149,875 (67.3)
Yes		72,920 (32.7)
Duration of NDH before T2DM diagnosis (days), median (IQR)		1,120 (648, 1,913)
HbA1c at identification/diagnosis (mmol/l), median (IQR)	43.0 (42.0, 44.0)	50.0 (47.0, 56.0)

*Abbreviations*: *IQR* Interquartile range, *SD* Standard deviation

### Inequalities in rates of NDH identification and T2DM diagnosis

**Fig. 1:**
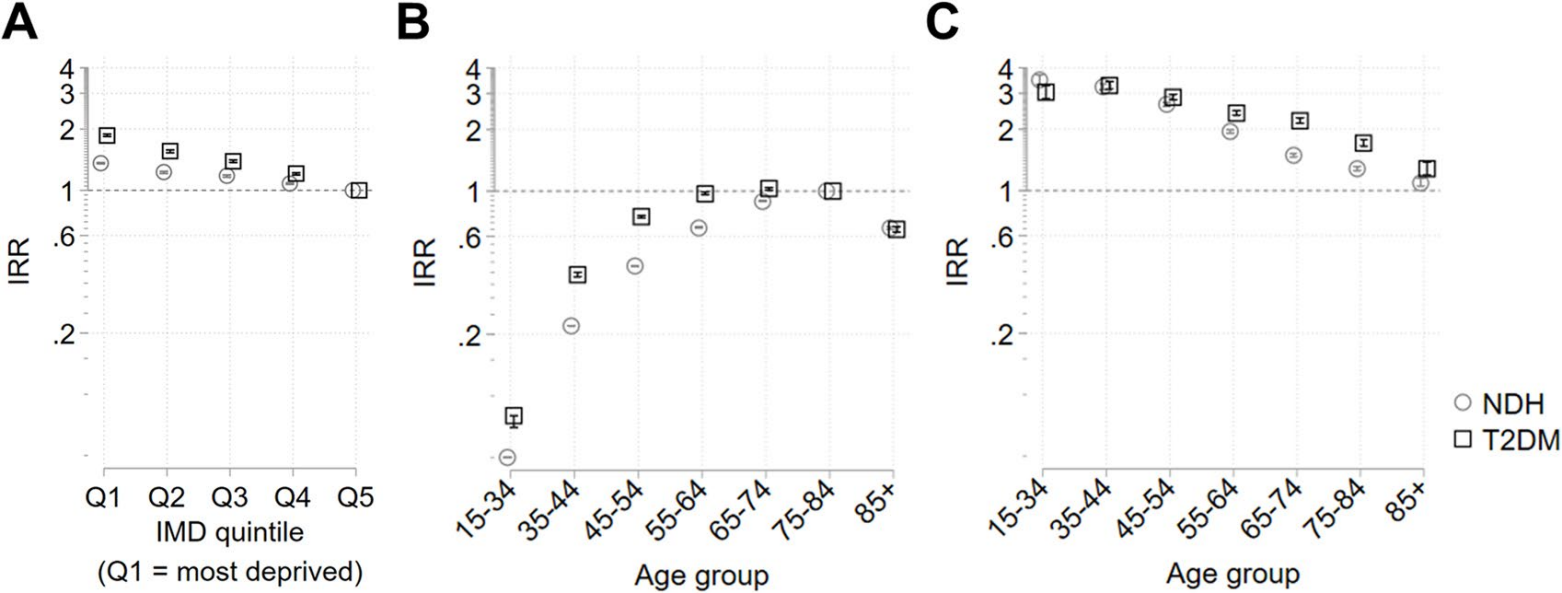
Deprivation- and age-related inequalities in NDH identification and T2DM diagnoses. Results from Poisson regression models plotted as incidence rate ratios (IRR) with 95% confidence intervals (CI). The reference group for deprivation is the least deprived group (quintile 5, Q5), and the reference group for age is 75–84 years. Horizontal dashed lines indicate IRR = 1 (no difference from reference group). Results for inequalities in 2019/20 NDH identification shown in grey with circle marker, results for 2019/20 T2DM diagnoses shown in black with square marker. **A** Inequalities by deprivation quintile. **B** Inequalities by age group. **C** Age-stratified inequalities between deprivation quintile 1 (Q1) and Q5. Results are also shown in Tables S1 – S4, along with the probability of the outcome in the base (reference) category

Compared to the least deprived areas, NDH recording rates increased with increasing deprivation, with the highest rate in the most deprived quintile (Q1 IRR 1.36 [1.35, 1.37]) (Fig. 1A, Table S1). However, inequalities in rates of T2DM diagnosis were wider than for NDH for each quintile (e.g.: Q1 IRR 1.86 [1.84, 1.89]).

Compared to people aged 75–84, NDH identification rates decreased with decreasing age, reaching an IRR 0.05 [0.05, 0.05] in the youngest age group (age 15–34) (Fig. 1B, Table S2). However, rates of T2DM diagnosis were slightly higher amongst those aged 65–74 (IRR 1.03 [1.01, 1.04]) compared to those aged 75–84, and decreased less sharply amongst younger age groups (IRR 0.08 [0.07, 0.08] age 15–34) suggesting an under identification of NDH relative to T2DM diagnosis rates amongst all age groups younger than 75–84.

Stratifying by age group showed that socioeconomic inequalities in rates of both NDH identification and T2DM diagnosis between the most (Q1) and least (Q5) deprived quintiles are greatest amongst those aged 15–34 and decreased monotonically with increasing age (Fig. 1C, Tables S3 & S4). However, inequalities in under identification of NDH (where inequalities in NDH identification are smaller than those in T2DM diagnosis) only occurred amongst groups aged 45 plus, with the greatest differences concentrated between age groups 55 to 84. There were similar patterns in age-stratified inequalities across intermediate levels of deprivation (Tables S3 & S4).

### Inequalities in not having previous NDH recorded prior to diagnosis with T2DM

**Fig. 2:**
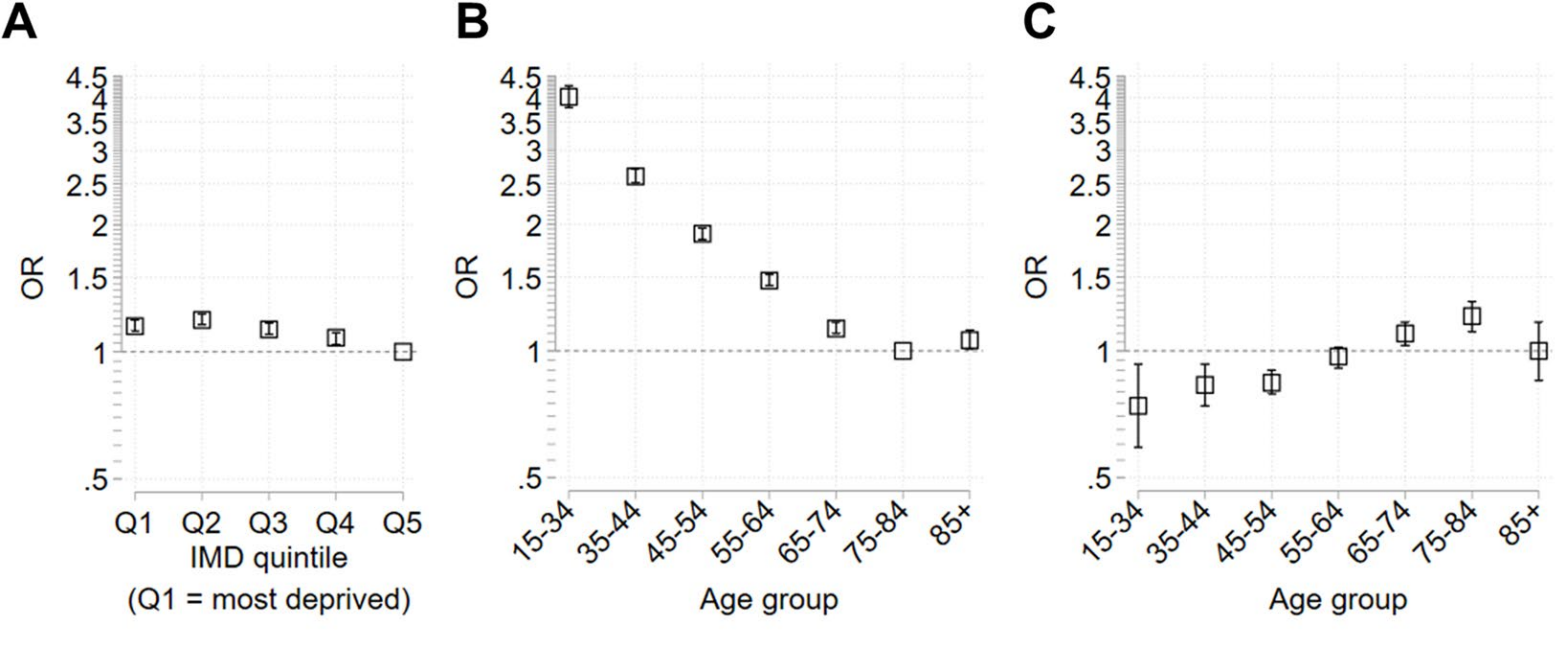
Inequalities in not having previous NDH recorded amongst people diagnosed with T2DM during 2019/20. Results from logistic regression models plotted as odds ratios (OR) with 95% confidence intervals (CI). The reference group for deprivation is the least deprived group (quintile 5, Q5), and the reference group for age is 75–84 years. Horizontal dashed lines indicate OR = 1 (no difference from reference group). **A** Inequalities by deprivation quintile. **B** Inequalities by age group. **C** Inequalities between deprivation quintile 1 (most deprived) and quintile 5 (reference group) stratified by age group. Full results also shown in Tables S5 – S7, along with the probability of the outcome in the base (reference) category

Although there was a large total population of people identified with NDH by 2019/20 (2,409,605), within those newly diagnosed with T2DM in 2019/20, the majority (149,875, 67.3%) had no previous NDH recorded (Table 1). Relative to people in the least deprived areas, there was increased odds of not having a previous NDH diagnosis for all deprivation quintiles, with the odds increasing with increasing deprivation up to Q2 (OR 1.19 [1.16, 1.23]) (Fig. 2A, Table S5). Age-related inequalities were wider than deprivation-related inequalities. Relative to people aged 75–84, younger age groups had increasing odds of not having previous NDH recorded up to the youngest age group (OR 4.02 [3.79, 4.27]) (Fig. 2B, Table S6). Stratifying by age group showed deprivation-related inequalities persisted amongst older adults (ages 65–84) (Fig. 2C, Table S7). However, there was evidence of lower odds of no previous NDH identification associated with increasing deprivation amongst younger age groups (under 55), with differences reaching statistical significance in the two most deprived quintiles.

### Inequalities in duration of previous NDH identification amongst people diagnosed with T2DM

**Fig. 3:**
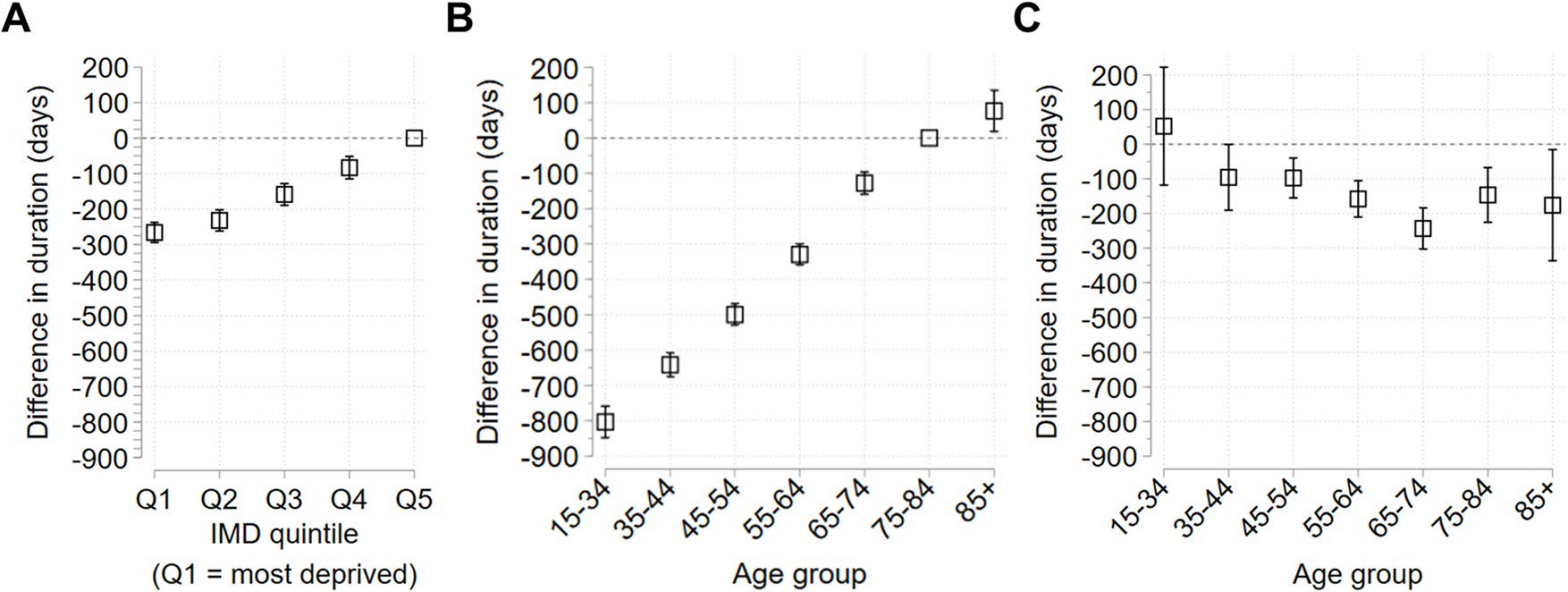
Inequalities in duration of previous NDH identification amongst people diagnosed with T2DM during 2019/20. Results from Poisson regression models used to compute mean difference in duration between groups. Mean difference in duration (in days) plotted with 95% confidence intervals (CI). The reference group for deprivation is the least deprived group (quintile 5, Q5), and the reference group for age is 75–84 years. Horizontal dashed lines indicate difference = 0 (no difference from reference group). **A** Inequalities by deprivation quintile. **B** Inequalities by age group. **C** Inequalities between deprivation quintile 1 (most deprived) and quintile 5 (reference group) stratified by age group. Full results also shown in Tables S8 – S10

Amongst those diagnosed with T2DM in 2019/20 who had a previous recording of NDH (72,920 out of 222,795), there were further inequalities in the duration of NDH. Compared to people in the least deprived areas, the duration of NDH decreased with increasing deprivation, reaching 266.17 [294.39, 237.96] days shorter amongst people in the most deprived areas (Fig. 3A, Table S8). Again, there were wider inequalities by age group. Compared to people aged 75–84, duration of previous NDH decreased with decreasing age, to 803.23 [848.03, 758.43] days shorter amongst people aged under 35 (Fig. 3B, Table S9). Stratification by age group showed increasing deprivation was associated with shorter NDH duration across most age groups, with differences reaching statistical significance for those aged 35 and over for Q1, age groups 35–84 for Q2, and age groups 35–74 for Q3 and Q4 (Fig. 3C, Table S10).

### Inequalities in severity (HbA1c value) at the time of NDH identification or T2DM diagnosis

A total of 68.9% (323,630) of individuals with newly identified NDH and 46.1% (102,605) of individuals with a new T2DM diagnosis had a HbA1c test result recorded at the time of their diagnosis (Figure S1). The odds of having a HbA1c test result recorded were higher in the most deprived groups, most younger age groups as well as those aged 85 plus, and amongst those with previously identified NDH, but the magnitudes of all associations were small (Table S11).

**Fig. 4:**
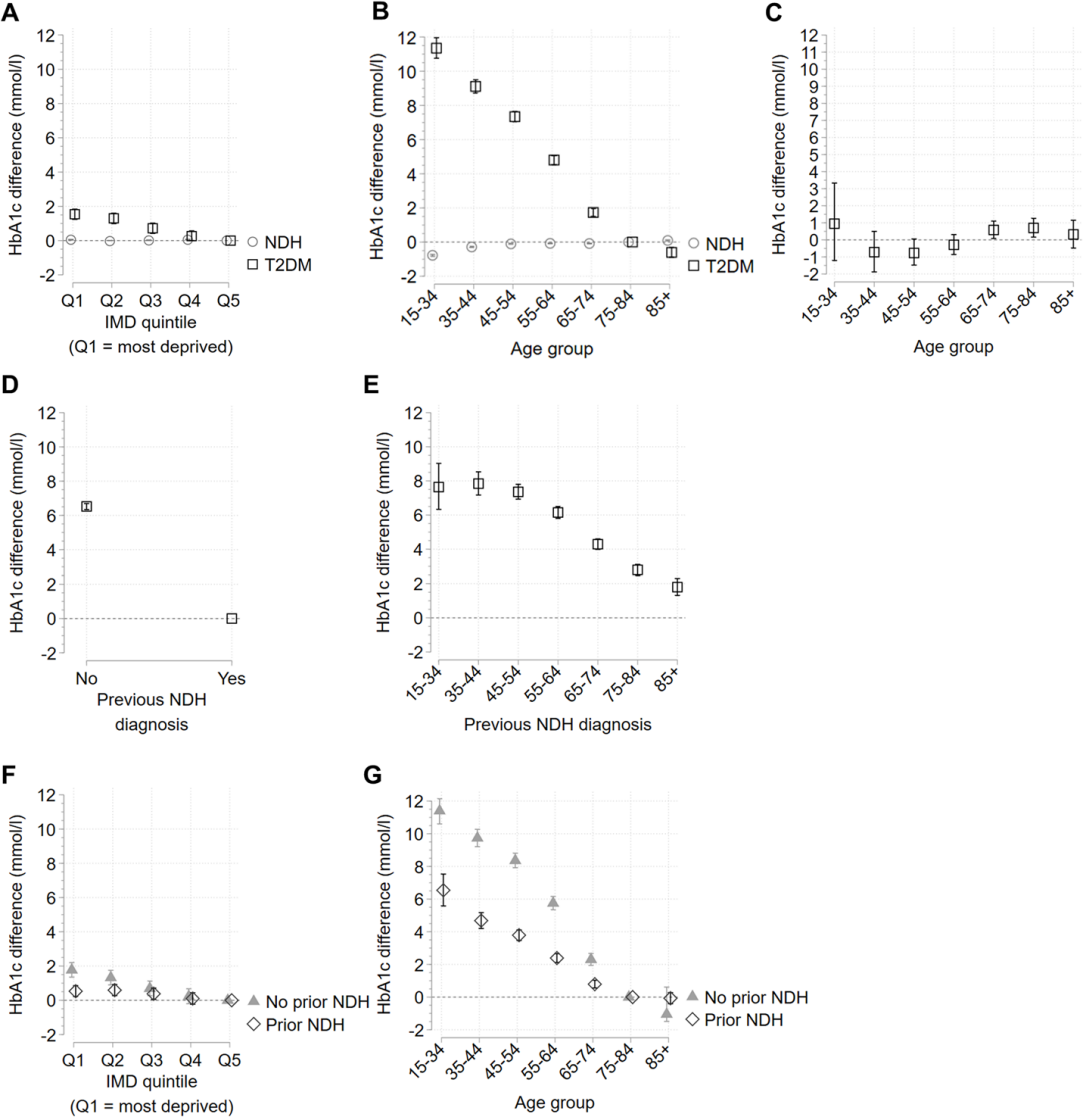
Inequalities in severity (HbA1c value) at the time of NDH identification or T2DM diagnosis. Results from linear regression models plotted as estimated difference in HbA1c (in mmol/l) between groups with 95% confidence intervals (CI). For the population with a new T2DM diagnosis, values were first log-transformed then the difference on a linear scale was computed after regression. The reference group for deprivation is the least deprived group (quintile 5, Q5), the reference group for age is 75–84 years, and the reference group for previous NDH identification is the presence of previous NDH Read code (Yes). Horizontal dashed lines indicate difference = 0 (no difference from reference group). Results for inequalities in 2019/20 NDH recording are shown in grey with a circle marker, results for 2019/20 T2DM diagnoses are shown in black with a square marker. **A** Inequalities by deprivation quintile. **B** Inequalities by age group. **C** Inequalities between deprivation quintile 1 (most deprived) and quintile 5 (reference group) stratified by age group. **D** Inequalities by occurrence or absence of a previous NDH record. **E** Inequalities by occurrence or absence of a previous NDH record stratified by age group. **F** Inequalities by deprivation quintile stratified by occurrence or absence of a previous NDH record. Results for no prior NDH record are shown in grey with a triangle marker, results for those with a prior NDH record are shown in black with a diamond marker **G** Inequalities by age group stratified by occurrence or absence of a previous NDH record. Results for no prior NDH record are shown in grey with a triangle marker, results for those with a prior NDH record are shown in black with a diamond marker. Full results also shown in Tables S12 – S18

For individuals with HbA1c results recorded at the time of T2DM diagnosis, HbA1c values increased with increasing deprivation up to 1.54 mmol/l [1.24, 1.84] higher for people living in the most compared to the least deprived areas (Fig. 4A, Table S12). Age-related inequalities were again larger, with HbA1c at T2DM diagnosis 11.35 mmol/l [10.76, 11.96] higher amongst people aged 15–34 compared to those aged 75–84 (Fig. 4B, Table S13). There were narrower deprivation-related inequalities in HbA1c at T2DM diagnosis when we stratified by age, but inequalities persisted within people aged 65–84 (Fig. 4C, Table S14). In contrast to HbA1c inequalities at the time of T2DM diagnosis, there were minimal age- or deprivation- related inequalities in HbA1c values at the time of NDH identification (Figs.  4A & B, Tables S12, S13 & S19).

### Association between previous NDH identification and severity at the time of T2DM diagnosis

Not having a previous record of NDH was associated with a 6.52 mmol/l [6.34, 6.69] higher HbA1c at the time of T2DM diagnosis compared to those with a previous record of NDH (Fig. 4D, Table S15). Stratifying by age showed that inequalities in HbA1c at T2DM diagnosis for people without a previous NDH record were present amongst each age group, with the widest inequalities amongst individuals aged under 55 (Fig. 4E, Table S16).

Stratifying the population by previous NDH identification and re-estimating deprivation-related and age-related inequalities in HbA1c at the time of T2DM diagnosis showed inequalities were generally smaller amongst those with a previous NDH record than amongst those without (Figs. 4F & G, Tables S17 & S18).

## Discussion

### Main findings

Using data from the National Diabetes Audit, we found NDH/intermediate hyperglycaemia is under identified relative to rates of T2DM diagnosis amongst people living in more deprived areas and amongst younger age groups in England. Deprivation-related inequalities persisted after stratification by age group, with inequalities concentrated amongst middle and older age groups (55–84). Despite high numbers of identification of new NDH cases over the past decade, most people newly diagnosed with T2DM had no previous record of NDH. Amongst people diagnosed with T2DM, people living in deprived areas and particularly younger people had higher odds of having no previous record of NDH.

There was additional inequality amongst those who had a previous NDH record before being diagnosed with T2DM, with a shorter recorded duration of NDH before T2DM diagnosis amongst people from more deprived areas and younger people. Analysis of HbA1c blood test results suggested there were minimal age- or deprivation-related inequalities in severity at the time of NDH identification, so together these results imply faster disease progression from NDH to T2DM amongst younger people and those living in more deprived areas. T2DM diagnosis severity was also higher amongst people living in more deprived areas, younger people, and people who had no previous NDH recorded. There were also greater age- and deprivation-related inequalities in T2DM severity amongst those with no NDH identified, highlighting multiple potential benefits of prior NDH case finding.

### Strengths and weaknesses

A strength of this study is the use of the National Diabetes Audit, a robust routinely collected dataset drawn from Read codes in primary care records covering over 99% of general practices in England [16]. The major primary care pay-for-performance scheme in England (QOF) has included T2DM targets since 2012/13, so we expect this to be very reliably coded [21]. While there is now an NDH QOF target, this was only introduced the year after the study period (2021/22) [21, 22] so there may be more variation in NDH coding between GP practices. Similarly, the Read code for NDH was only introduced recently (2016) [23], so NDH cases prior to this are based on derived codes and may be less reliable.

Another limitation of the National Diabetes Audit is the high level of missing data for HbA1c blood test results, which may have introduced some bias. Although we found younger people and those living in more deprived areas have slightly higher odds of recorded HbA1c values, associations tended to be small in magnitude so our overall conclusions about inequalities in severity of T2DM are likely to hold. Only the analyses of inequalities in the severity of T2DM at the time of diagnosis are affected by missing HbA1c data, as the remainder of our analyses use the recording of clinical Read codes for NDH and T2DM in patient records rather than recorded HbA1c values. We were also unable to obtain information on medications prescribed to individuals which, if already being taken at the point of HbA1c measurement, could have impacted the recorded HbA1c values. The dataset also lacks information about individual socioeconomic status, so we used area-level deprivation as a proxy measure. Given there is substantial heterogeneity in individual socioeconomic status within each neighbourhood [24], our results likely underestimate individual-level inequalities.

Attempts to understand inequalities in NDH and T2DM prevalence are complicated by sociodemographic inequalities in rates of conversion from NDH to T2DM and mortality rates. A strength of our approach is the focus on incident cases of NDH and T2DM, which also gives a clear indication of newly occurring inequalities, separated from cumulative inequalities from previous years. However, our retrospective analysis of previous NDH identification amongst those newly diagnosed with T2DM also adds important information about total inequalities in NDH identification aggregated over time. In all analyses, we consider rates of T2DM diagnosis in each sociodemographic group to approximate true underlying rates of T2DM per population. However, there are known age- and deprivation-related inequalities in the under diagnosis of T2DM [25], suggesting our results likely underestimate true inequalities in the under identification of NDH.

Finally, because we are using data from 2019/20, our results likely underestimate current inequalities. Since the onset of the COVID-19 pandemic, there has been widespread disruption to and increased pressure on NHS healthcare services, and both rates of NDH identification and health checks for those identified with existing NDH fell during 2020/21 [12]. Given that there are fewer general practice doctors per population in more deprived areas [26], it is likely that these impacts have been felt disproportionately in areas of higher deprivation, so inequalities are likely to have widened.

### Comparison to literature

Our results are consistent with existing evidence that NDH is under identified amongst older adults living in more deprived areas in England [13]. Importantly, here we consider adults of all age groups, adding new evidence that deprivation-related inequalities tend to be concentrated amongst middle and older age groups. More broadly, our evidence of inequalities at the earliest stage of the NHS DPP care pathway (NDH identification) adds to the existing evidence of deprivation-related inequalities at each subsequent stage of the pathway [8–10].

The findings of our NDH duration analyses, that younger age groups have faster progression from NDH identification to T2DM and higher HbA1c at the time of T2DM diagnosis concur with previous evidence [4, 13–15]. Here we add to this evidence, by newly finding that NDH is also under identified relative to T2DM diagnosis in all age groups under 75, with the greatest under identification amongst under 35s. Missed opportunities for T2DM delay or prevention amongst this group are of particular concern given that rates of young-onset (aged under 40) T2DM are increasing more quickly than amongst any other age group, and younger people would be expected to live longer with T2DM increasing the life-course burden of disease for both individuals and health systems [11]. There is also increasing evidence that disease progression is more severe and is accompanied by higher risks of T2DM-related complications and premature mortality for people with young-onset T2DM [11]. Young-onset T2DM is more common amongst women (compared to men) and amongst some ethnic minority groups (compared to White British) in England [11, 27], so our results also have implications for gender and ethnic inequalities.

At the other end of the age range, previous work has raised questions about whether NDH identification is always beneficial amongst older adults [28]. Given that NDH is not itself a symptomatic medical condition, that rates of conversion from NDH to T2DM decrease with increasing age, and that the risk of T2DM complications rises with increasing T2DM duration, the value of preventing or delaying T2DM is lower amongst those with shorter life expectancy at the time of NDH identification. Previous authors have argued that when this is balanced against the potential harms of weight loss amongst older adults and potential psychological distress, NDH identification or T2DM prevention interventions may not always be in older peoples’ best interests [28]. In this context, it is notable that almost 20% of new NDH records in 2019/20 were amongst people aged 75 or above.

### Meaning and implications

We found evidence suggesting NDH/intermediate hyperglycaemia is under identified relative to rates of T2DM diagnosis amongst all age groups under 75 and all levels of deprivation compared to the least deprived areas. There were age- and deprivation-related gradients, with the greatest under identification of NDH amongst the youngest individuals. Our results suggest there are currently many missed opportunities for interventions to prevent or delay T2DM, so have implications for both population-level efficiency and equity of NHS T2DM prevention efforts.

Despite substantial increases in NDH identification and a now large population with recorded NDH, most individuals who received a T2DM diagnosis in 2019/20 did not have a previous record of NDH. Given that HbA1c levels are not constantly monitored in the general population, we cannot tell whether the remaining individuals progressed directly from normal glycaemic control to diabetes or whether they had glycaemic levels within the NDH range at some point, but this was not detected because their HbA1c levels were not tested. Either way, our results suggest that existing strategies to identify people with NDH may not actually be picking up people at the highest risk of developing T2DM. Our results, together with previous evidence of rates of conversion from NDH to T2DM [12–15], suggest this may be partly driven by high levels of NDH case finding amongst older adults, who are less likely to develop T2DM. There therefore remains a debate over whether targeted high-risk or population-wide prevention strategies are the most effective approach to diabetes prevention.

One factor that may contribute to inequalities in NDH identification is the use of NHS health checks for NDH detection [29]. Health check appointments are offered every five years, but only from age 40 and above [29]. Given that diagnosis rates of diabetes are increasing fastest amongst those under 40 [11], this age criterion may result in important missed opportunities for T2DM prevention. Similarly, attendance at health check appointments is lower amongst all age groups under 65–69, and attendance rates decrease with increasing deprivation [30]. Increasing equity of attendance at NHS health checks could therefore help address these inequalities.

We suggest that to increase the equity of T2DM prevention opportunities, more active NDH case-finding is required amongst younger people and those living in more deprived areas. However, attention should also be paid to our results indicating that age- and deprivation-related inequalities in new T2DM diagnoses persist even amongst those with a previous record of NDH, along with previous evidence of sociodemographic inequalities in outcomes even amongst those referred to the DPP [9, 10].

### Unanswered questions and future research

Here we focused on deprivation and age-related inequalities, but further research is needed to investigate additional dimensions of inequality such as gender and ethnicity. It will also be important to understand which factors drive the observed inequalities, and to design and evaluate interventions to address these inequalities.

### Supplementary Information

Supplementary Material 1.

## Data Availability

The data that support the findings of this study are available upon application from NHS Digital, but restrictions apply to the availability of these data, which were used under license for the current study (reference: DARS-NIC-196221-K4K3Y), and so are not publicly available. The use of this data is subject to various output rules, including rounding count values to the nearest 5.

## Abbreviations

CI: Confidence intervals
DPP: Diabetes Prevention Programme
HbA1c: Glycated haemoglobin (A1c)
IMD: Index of multiple deprivation
IQR: Interquartile range
IRR: Incidence rate ratios
LSOA: Lower-layer super output area
NDA: National Diabetes Audit
NDH: Non-diabetic hyperglycaemia
NHS: National Health Service
OR: Odds ratios
QOF: Quality and Outcomes Framework
SD: Standard deviation
T2DM: Type 2 diabetes mellitus
