# Supplementary material for: Age- and deprivation-related inequalities in identification of people at high risk of type 2 diabetes in England
Source: BMC Public Health. 2024 Aug 10;24:2166. doi: 10.1186/s12889-024-19571-x (PMC11316385; doi:10.1186/s12889-024-19571-x)
Supplement: Supplementary file 1 — Supplementary Material 1. [file 12889_2024_19571_MOESM1_ESM.docx]

**Supplementary material**

**Figure S1 – Flow chart**


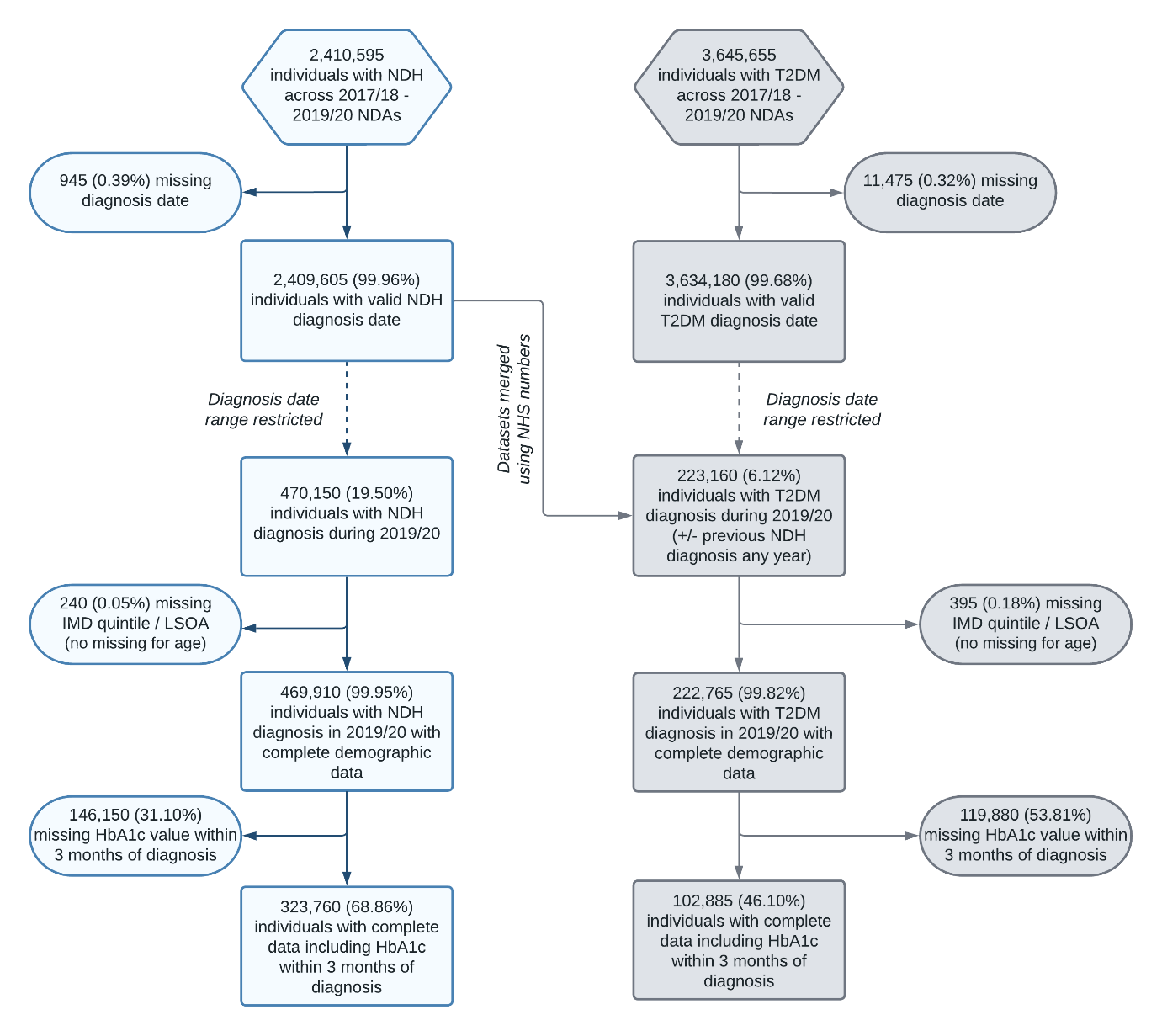


**Table S1 – Deprivation-related inequalities in rates of new NDH identification and T2DM diagnosis**

|  | Incident Rate Ratio | | |
| --- | --- | --- | --- |
|  | NDH | T2DM | |
| **IMD quintile** |  |  | |
| Q1 (most deprived) | 1.36^***^ | 1.86^***^ | |
|  | [1.35 - 1.37] | [1.84 - 1.89] | |
| Q2 | 1.23^***^ | 1.56^***^ | |
|  | [1.21 - 1.24] | [1.53 - 1.58] | |
| Q3 | 1.18^***^ | 1.39^***^ | |
|  | [1.16 - 1.19] | [1.37 - 1.41] | |
| Q4 | 1.08^***^ | 1.21^***^ | |
|  | [1.07 - 1.09] | [1.19 - 1.22] | |
| Q5 (least deprived) | 1.00 | 1.00 | |
|  | -- | -- | |
| Observations | 40762665 | 42700925 | |
| Probability of being newly identified with NDH in the least deprived quintile (p0) = 0.010  Probability of being newly diagnosed with T2DM in the least deprived quintile (p0) = 0.004  Notes: ^*^ *p* < 0·05, ^**^ *p* < 0·01, ^***^ *p* < 0·001 | | |  |

**Table S2 – Age-related inequalities in rates of new identification of NDH and T2DM diagnosis**

|  | Incident Rate Ratio | |
| --- | --- | --- |
|  | NDH | T2DM |
| **Age group** |  |  |
| 15-34 | 0.05^***^ | 0.08^***^ |
|  | [0.05 - 0.05] | [0.07 - 0.08] |
| 35-44 | 0.22^***^ | 0.39^***^ |
|  | [0.22 - 0.22] | [0.38 - 0.40] |
| 45-54 | 0.43^***^ | 0.75^***^ |
|  | [0.43 - 0.43] | [0.74 - 0.76] |
| 55-64 | 0.66^***^ | 0.97^***^ |
|  | [0.66 - 0.67] | [0.96 - 0.99] |
| 65-74 | 0.89^***^ | 1.03^***^ |
|  | [0.89 - 0.90] | [1.01 - 1.04] |
| 75-84 | 1.00 | 1.00 |
|  | -- | -- |
| 85+ | 0.66^***^ | 0.65^***^ |
|  | [0.65 - 0.67] | [0.63 - 0.67] |
| Observations | 40762665 | 42700925 |
| Probability of being newly identified with NDH in the age category 75-84 (p0) = 0.030  Probability of being newly diagnosed with T2DM in the age category 75-84 (p0) = 0.010  Notes: ^*^ *p* < 0·05, ^**^ *p* < 0·01, ^***^ *p* < 0·001 | | |

**Figure S2 – Population age structures**

**A. Populations by age and IMD quintile B. Pop. with pre-existing NDH C. Populations with pre-existing T2DM**

**
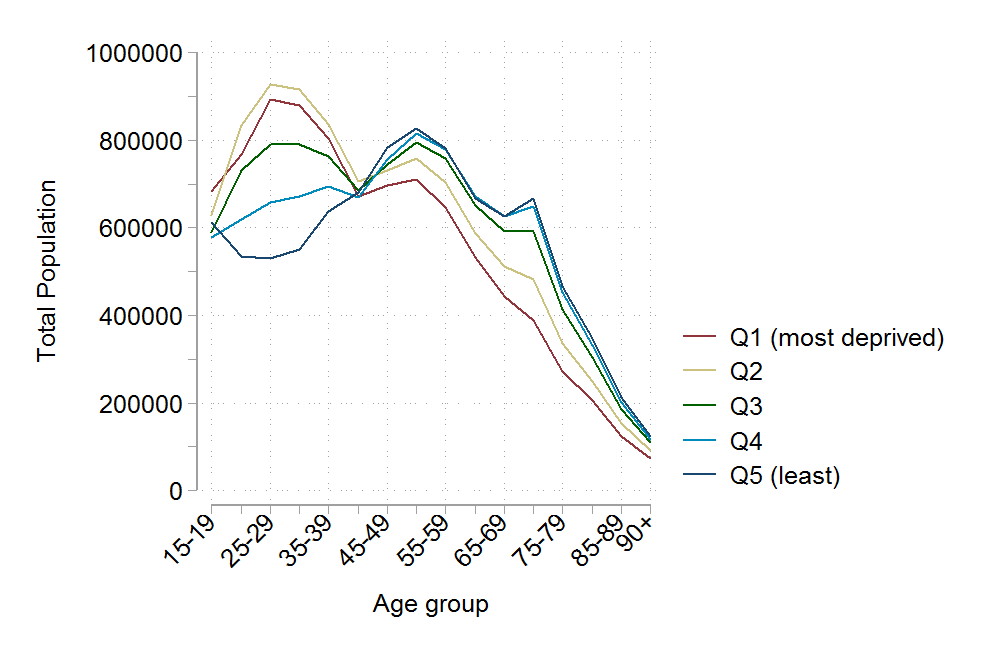

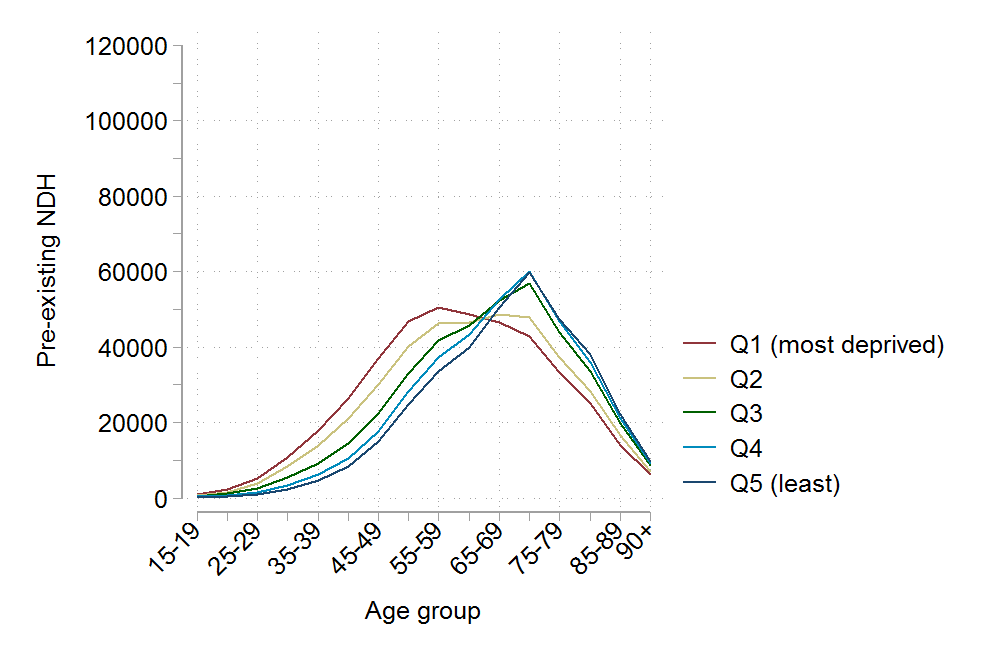

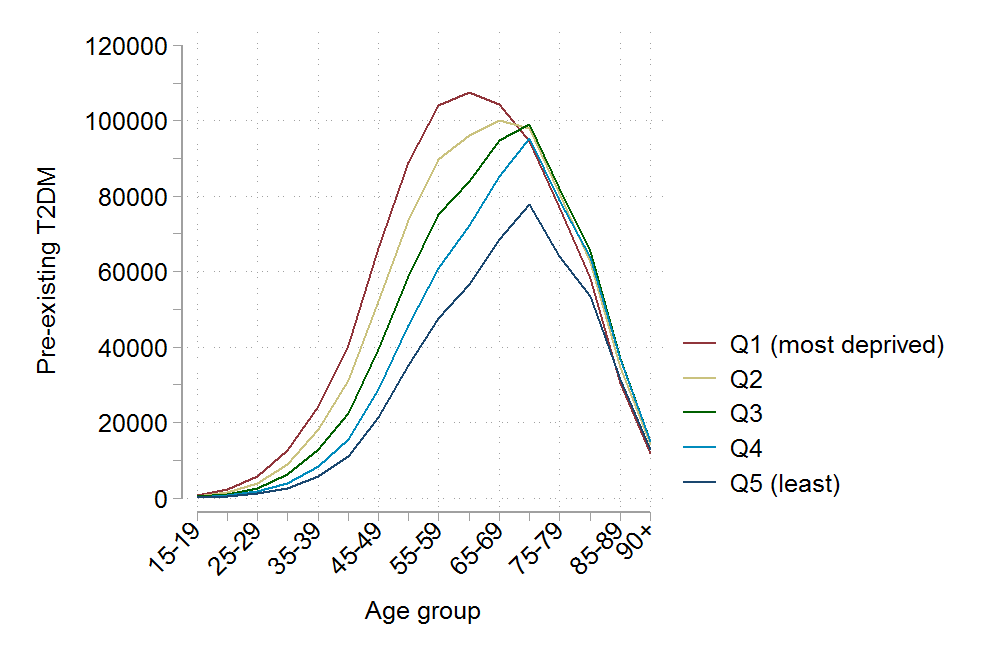
**

**D. Populations at-risk of NDH E. Populations at-risk of T2DM**

**
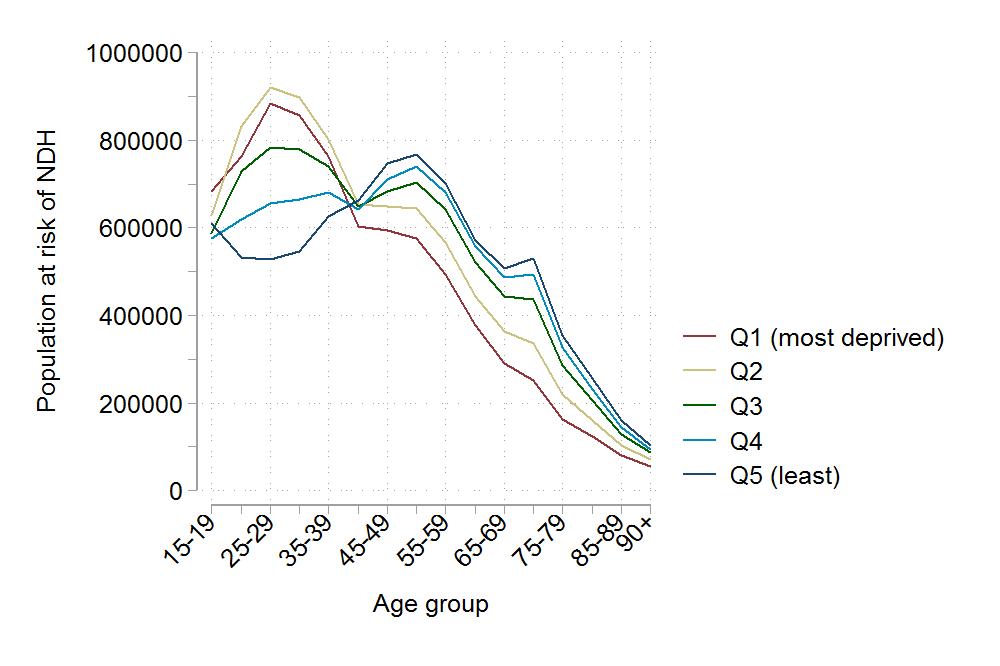

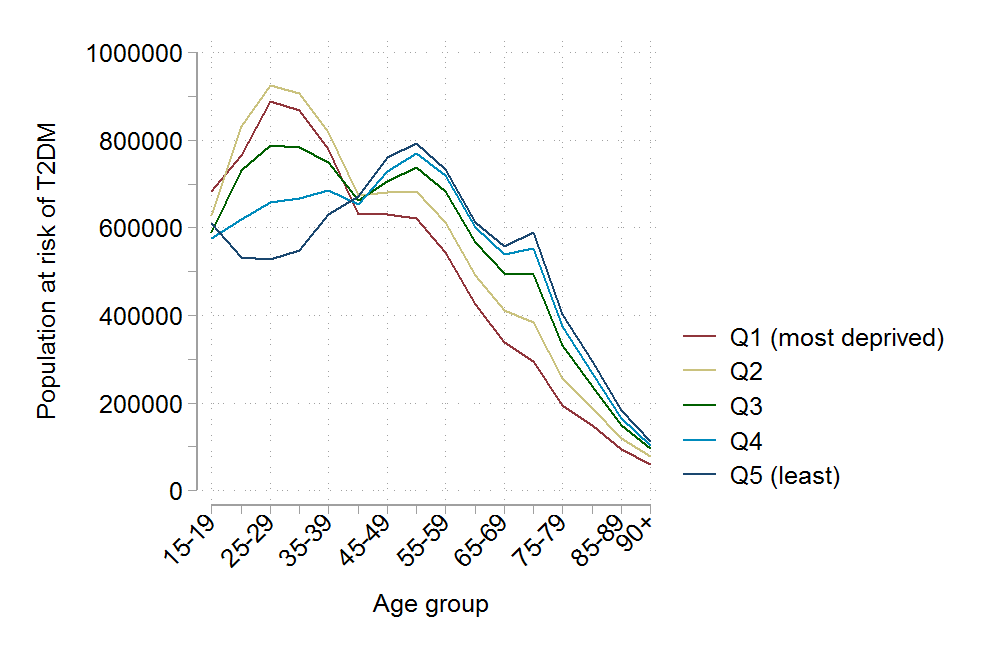
**

**Table S3 – Age-stratified IMD-related inequalities in rates of new identification of NDH**

|  | Incident Rate Ratio | | | | | | |
| --- | --- | --- | --- | --- | --- | --- | --- |
|  | 15-34 | 35-44 | 45-54 | 55-64 | 65-74 | 75-84 | 85+ |
| **IMD Quintile** |  |  |  |  |  |  |  |
| Q1 (most deprived) | 3.49^***^ | 3.23^***^ | 2.65^***^ | 1.95^***^ | 1.49^***^ | 1.28^***^ | 1.09^***^ |
|  | [3.30 - 3.69] | [3.12 - 3.34] | [2.59 - 2.71] | [1.91 - 1.99] | [1.46 - 1.52] | [1.25 - 1.31] | [1.05 - 1.15] |
| Q2 | 2.82^***^ | 2.55^***^ | 2.13^***^ | 1.63^***^ | 1.32^***^ | 1.21^***^ | 1.04 |
|  | [2.66 - 2.99] | [2.46 - 2.64] | [2.08 - 2.18] | [1.60 - 1.66] | [1.29 - 1.34] | [1.19 - 1.24] | [1.00 - 1.09] |
| Q3 | 2.15^***^ | 1.82^***^ | 1.64^***^ | 1.39^***^ | 1.21^***^ | 1.16^***^ | 1.11^***^ |
|  | [2.02 - 2.28] | [1.76 - 1.89] | [1.60 - 1.68] | [1.36 - 1.41] | [1.19 - 1.23] | [1.13 - 1.18] | [1.06 - 1.15] |
| Q4 | 1.65^***^ | 1.36^***^ | 1.28^***^ | 1.16^***^ | 1.09^***^ | 1.06^***^ | 1.03 |
|  | [1.55 - 1.76] | [1.30 - 1.41] | [1.25 - 1.31] | [1.14 - 1.18] | [1.07 - 1.10] | [1.04 - 1.09] | [0.99 - 1.08] |
| Q5 (least deprived) | 1.00 | 1.00 | 1.00 | 1.00 | 1.00 | 1.00 | 1.00 |
|  | -- | -- | -- | -- | -- | -- | -- |
| Observations | 14077215 | 6825230 | 6817510 | 5554900 | 4140050 | 2324910 | 1022850 |
| Probability of being newly identified with NDH in the least deprived quintile (p0) | 0.001 | 0.003 | 0.008 | 0.015 | 0.023 | 0.027 | 0.019 |
| Notes: ^*^ *p* < 0·05, ^**^ *p* < 0·01, ^***^ *p* < 0·001 | | | | | | | |

**Table S4 – Age-stratified IMD-related inequalities in rates of new diagnosis of T2DM**

|  | Incident Rate Ratio | | | | | | |
| --- | --- | --- | --- | --- | --- | --- | --- |
|  | 15-34 | 35-44 | 45-54 | 55-64 | 65-74 | 75-84 | 85+ |
| **IMD Quintile** |  |  |  |  |  |  |  |
| Q1 (most deprived) | 3.04^***^ | 3.28^***^ | 2.87^***^ | 2.40^***^ | 2.20^***^ | 1.71^***^ | 1.28^***^ |
|  | [2.82 - 3.28] | [3.14 - 3.43] | [2.79 - 2.96] | [2.34 - 2.47] | [2.14 - 2.27] | [1.65 - 1.78] | [1.19 - 1.38] |
| Q2 | 2.24^***^ | 2.43^***^ | 2.25^***^ | 1.90^***^ | 1.79^***^ | 1.57^***^ | 1.32^***^ |
|  | [2.07 - 2.42] | [2.32 - 2.55] | [2.18 - 2.32] | [1.85 - 1.95] | [1.74 - 1.85] | [1.51 - 1.64] | [1.23 - 1.42] |
| Q3 | 1.81^***^ | 1.91^***^ | 1.75^***^ | 1.54^***^ | 1.47^***^ | 1.36^***^ | 1.16^***^ |
|  | [1.67 - 1.96] | [1.82 - 2.00] | [1.69 - 1.80] | [1.49 - 1.58] | [1.43 - 1.51] | [1.31 - 1.41] | [1.08 - 1.24] |
| Q4 | 1.33^***^ | 1.34^***^ | 1.32^***^ | 1.23^***^ | 1.27^***^ | 1.25^***^ | 1.11^***^ |
|  | [1.22 - 1.46] | [1.28 - 1.42] | [1.28 - 1.36] | [1.20 - 1.27] | [1.23 - 1.31] | [1.21 - 1.30] | [1.04 - 1.19] |
| Q5 (least deprived) | 1.00 | 1.00 | 1.00 | 1.00 | 1.00 | 1.00 | 1.00^***^ |
|  | -- | -- | -- | -- | -- | -- | -- |
| Observations | 14130430 | 6957875 | 7113260 | 5988625 | 4658500 | 2694505 | 1157730 |
| Probability of being newly diagnosed with T2DM in the least deprived quintile (p0) | 0.0004 | 0.002 | 0.004 | 0.006 | 0.007 | 0.007 | 0.005 |
| Notes: ^*^ *p* < 0·05, ^**^ *p* < 0·01, ^***^ *p* < 0·001 | | | | | | | |

**Table S5 – IMD-related inequalities in the odds of not having NDH previously recorded**

|  | Odds ratio  (No NDH previously recorded) |
| --- | --- |
| **IMD quintile** |  |
| Q1 (most deprived) | 1.15^***^ |
|  | [1.12 - 1.19] |
| Q2 | 1.19^***^ |
|  | [1.16 - 1.23] |
| Q3 | 1.13^***^ |
|  | [1.10 - 1.17] |
| Q4 | 1.08^***^ |
|  | [1.04 - 1.11] |
| Q5 (least deprived) | 1.00 |
|  | -- |
| Observations | 222795 |
| Probability of not having NDH previously recorded in the least deprived quintile (p0) = 0.647  Notes: ^*^ *p* < 0·05, ^**^ *p* < 0·01, ^***^ *p* < 0·001  The relative risk can be calculated using the reported odds ratios alongside the probability of the outcome in the base (reference) category p0, via the following formula: Relative risk=odds ratio/(1−p0+(p0×odds ratio))^1^. | |

**Table S6 – Age-related inequalities in the odds of not having NDH previously recorded**

|  | Odds ratio  (No NDH previously recorded) |
| --- | --- |
| **Age group** |  |
| 15-34 | 4.02^***^ |
|  | [3.79 - 4.27] |
| 35-44 | 2.60^***^ |
|  | [2.51 - 2.71] |
| 45-54 | 1.90^***^ |
|  | [1.84 - 1.96] |
| 55-64 | 1.47^***^ |
|  | [1.43 - 1.52] |
| 65-74 | 1.13^***^ |
|  | [1.10 - 1.17] |
| 75-84 | 1.00 |
|  | -- |
| 85+ | 1.06^*^ |
|  | [1.01 - 1.12] |
| Observations | 222795 |
| Probability of not having NDH previously recorded in the age category 75-84 (p0) = 0.573  Notes: ^*^ *p* < 0·05, ^**^ *p* < 0·01, ^***^ *p* < 0·001 | |

**Table S7 – Age-stratified IMD-related inequalities in the odds of not having NDH previously recorded**

|  | Odds Ratio (No NDH previously recorded) | | | | | | |
| --- | --- | --- | --- | --- | --- | --- | --- |
|  | 15-34 | 35-44 | 45-54 | 55-64 | 65-74 | 75-84 | 85+ |
| **IMD Quintile** |  |  |  |  |  |  |  |
| Q1 (most deprived) | 0.74^**^ | 0.83^***^ | 0.84^***^ | 0.97 | 1.10^**^ | 1.21^***^ | 1.00 |
|  | [0.59 - 0.93] | [0.74 - 0.93] | [0.79 - 0.90] | [0.91 - 1.02] | [1.03 - 1.17] | [1.11 - 1.31] | [0.85 - 1.17] |
| Q2 | 0.77* | 0.87^*^ | 0.95 | 1.01 | 1.16^***^ | 1.31^***^ | 1.30^***^ |
|  | [0.61 - 0.97] | [0.77 - 0.97] | [0.88 - 1.01] | [0.95 - 1.07] | [1.09 - 1.23] | [1.22 - 1.42] | [1.12 - 1.50] |
| Q3 | 0.80 | 0.96 | 0.94 | 1.03 | 1.13^***^ | 1.19^***^ | 1.12 |
|  | [0.63 - 1.01] | [0.85 - 1.08] | [0.88 - 1.01] | [0.97 - 1.09] | [1.07 - 1.21] | [1.11 - 1.29] | [0.98 - 1.30] |
| Q4 | 0.87 | 0.97 | 1.01 | 1.00 | 1.10^**^ | 1.16^***^ | 1.11 |
|  | [0.67 - 1.13] | [0.85 - 1.11] | [0.94 - 1.09] | [0.94 - 1.06] | [1.03 - 1.17] | [1.08 - 1.26] | [0.97 - 1.28] |
| Q5 (least deprived) | 1.00 | 1.00 | 1.00 | 1.00 | 1.00 | 1.00 | 1.00 |
|  | -- | -- | -- | -- | -- | -- | -- |
| Observations | 10260 | 26070 | 51195 | 56045 | 46105 | 25910 | 7220 |
| Probability of not having NDH previously recorded in the least deprived quintile (p0) | 0.873 | 0.796 | 0.733 | 0.665 | 0.580 | 0.535 | 0.564 |
| Notes: ^*^ *p* < 0·05, ^**^ *p* < 0·01, ^***^ *p* < 0·001 | | | | | | | |

**Table S8 – IMD-related inequalities in the duration of previous NDH recording**

|  | Difference in duration  (days) |
| --- | --- |
| **IMD quintile** |  |
| Q1 (most deprived) | -266.17^***^ |
|  | [-294.39 - -237.96] |
| Q2 | -232.24^***^ |
|  | [-262.06 - -202.42] |
| Q3 | -158.90^***^ |
|  | [-189.71 - -128.09] |
| Q4 | -83.33^***^ |
|  | [-115.06 - -51.59] |
| Q5 (least deprived) | 0.00 |
|  | -- |
| Observations | 72920 |
| Notes: ^*^ *p* < 0·05, ^**^ *p* < 0·01, ^***^ *p* < 0·001 | |

**Table S9 – Age-related inequalities in the duration of previous NDH recording**

|  | Difference in duration  (days) |
| --- | --- |
| **Age group** |  |
| 15-34 | -803.23^***^ |
|  | [-848.03 - -758.43] |
| 35-44 | -641.47^***^ |
|  | [-675.19 - -607.75] |
| 45-54 | -499.21^***^ |
|  | [-528.97 - -469.45] |
| 55-64 | -329.96^***^ |
|  | [-359.56 - -300.36] |
| 65-74 | -127.75^***^ |
|  | [-159.30 - -96.19] |
| 75-84 | 0.00 |
|  | -- |
| 85+ | 76.54^**^ |
|  | [18.21 - 134.88] |
| Observations | 72920 |
| Notes: ^*^ *p* < 0·05, ^**^ *p* < 0·01, ^***^ *p* < 0·001 | |

**Table S10 – Age-stratified IMD-related inequalities in the duration of previous NDH recording**

|  | Difference in duration  (days) | | | | | | |
| --- | --- | --- | --- | --- | --- | --- | --- |
|  | 15-34 | 35-44 | 45-54 | 55-64 | 65-74 | 75-84 | 85+ |
| **IMD Quintile** |  |  |  |  |  |  |  |
| Q1 (most) | 52.17 | -95.68^*^ | -97.37^***^ | -157.83^***^ | -243.35^***^ | -146.45^***^ | -176.13^*^ |
|  | [-118.19 - 222.52] | [-190.62 –  -0.73] | [-154.99 –  -39.76] | [-210.27 –  -105.40] | [-302.71 –  -183.98] | [-225.59 –  -67.31] | [-336.59 –  -15.68] |
| Q2 | -38.25 | -128.84^**^ | -128.79^***^ | -144.26^***^ | -182.65^***^ | -133.68^***^ | -109.05 |
|  | [-210.04 - 133.53] | [-225.29 –  -32.39] | [-187.77 –  -69.80] | [-198.75 –  -89.77] | [-247.71 –  -117.59] | [-212.98 –  -54.37] | [-277.69 - 59.59] |
| Q3 | 28.28 | -142.21^**^ | -95.54^*^ | -86.61^*^ | -145.07^***^ | -77.91 | -147.97 |
|  | [-155.86 - 212.42] | [-242.76 –  -41.66] | [-158.59 –  -32.49] | [-143.18 –  -30.05] | [-208.18 –  -81.97] | [-156.46 - 0.64] | [-308.57 - 12.63] |
| Q4 | 21.68 | -35.25^*^ | -52.90^*^ | -40.66^*^ | -117.65^***^ | -34.65 | -81.58^*^ |
|  | [-170.58 - 213.94] | [-149.74 - 79.25] | [-119.65 - 13.84] | [-99.87 - 18.54] | [-179.17 –  -56.14] | [-112.25 - 42.94] | [-241.55 - 78.39] |
| Q5 (least) | 0.00 | 0.00 | 0.00 | 0.00 | 0.00 | 0.00 | 0.00 |
|  | -- | -- | -- | -- | -- | -- | -- |
| Observations | 1600 | 5790 | 14420 | 18800 | 18285 | 11050 | 2975 |
| Notes: ^*^ *p* < 0·05, ^**^ *p* < 0·01, ^***^ *p* < 0·001 | | | | | | | |

**Table S11 – Inequalities in odds of having an HbA1c value recorded at the time of NDH identification or T2DM diagnosis**

|  | NDH recorded | T2DM diagnosis |
| --- | --- | --- |
|  |  |  |
| **IMD quintile** |  |  |
| Q1 (most deprived) | 1.06^***^ | 1.11^***^ |
|  | [1.04 - 1.08] | [1.07 - 1.14] |
| Q2 | 0.94^***^ | 1.09^***^ |
|  | [0.92 - 0.96] | [1.06 - 1.12] |
| Q3 | 0.99 | 1.06^***^ |
|  | [0.97 - 1.01] | [1.03 - 1.09] |
| Q4 | 0.94^***^ | 1.01 |
|  | [0.92 - 0.96] | [0.98 - 1.04] |
| Q5 (least deprived) | 1.00 | 1.00 |
|  | -- | -- |
| **Age group** |  |  |
| 15-34 | 0.86^***^ | 1.09^***^ |
|  | [0.83 - 0.88] | [1.04 - 1.14] |
| 35-44 | 1.03^*^ | 1.08^***^ |
|  | [1.00 - 1.06] | [1.04 - 1.12] |
| 45-54 | 1.08^***^ | 1.03^*^ |
|  | [1.05 - 1.10] | [1.00 - 1.06] |
| 55-64 | 1.03^***^ | 0.99 |
|  | [1.01 - 1.06] | [0.96 - 1.02] |
| 65-74 | 1.02 | 0.96^*^ |
|  | [1.00 - 1.04] | [0.94 - 0.99] |
| 75-84 | 1.00 | 1.00 |
|  | -- | -- |
| 85+ | 1.06^***^ | 1.19^***^ |
|  | [1.03 - 1.10] | [1.13 - 1.25] |
| **Previous NDH** |  |  |
| No | -- | 0.96^***^ |
|  |  | [0.94 - 0.98] |
| Yes | -- | 1.00 |
|  |  | -- |
|  |  |  |
| Observations | 469910 | 222795 |
| Notes: ^*^ *p* < 0·05, ^**^ *p* < 0·01, ^***^ *p* < 0·001 | | |

**Table S12 – Deprivation-related inequalities in HbA1c value at the time of NDH identification or T2DM diagnosis**

|  | Difference in HbA1c (mmol/l) -  NDH recorded | Difference in HbA1c (mmol/l) -  T2DM diagnosis |
| --- | --- | --- |
| **IMD quintile** |  |  |
| Q1 (most deprived) | 0.05^***^ | 1.54^***^ |
|  | [0.03 - 0.07] | [1.24-1.84] |
| Q2 | -0.03^*^ | 1.3^***^ |
|  | [-0.05 - -0.00] | [0.99-1.60] |
| Q3 | 0.01 | 0.72^***^ |
|  | [-0.01 - 0.04] | [0.42-1.02] |
| Q4 | 0.05^***^ | 0.26 |
|  | [0.03 - 0.08] | [-0.05-0.57] |
| Q5 (least deprived) | 0.00 | 0.00 |
|  | -- | -- |
| Observations | 323630 | 102605 |
| Notes: ^*^ *p* < 0·05, ^**^ *p* < 0·01, ^***^ *p* < 0·001 | | |

**Table S13 – Age-related inequalities in HbA1c value at the time of NDH identification or T2DM diagnosis**

|  | Difference in HbA1c (mmol/l) -  NDH recorded | Difference in HbA1c (mmol/l) -  T2DM diagnosis |
| --- | --- | --- |
| **Age group** |  |  |
| 15-34 | -0.78^***^ | 11.35^***^ |
|  | [-0.84 - -0.73] | [10.76-11.96] |
| 35-44 | -0.29^***^ | 9.11^***^ |
|  | [-0.32 - -0.25] | [8.72-9.50] |
| 45-54 | -0.11^***^ | 7.34^***^ |
|  | [-0.13 - -0.08] | [7.04-7.65] |
| 55-64 | -0.08^***^ | 4.8^***^ |
|  | [-0.10 - -0.06] | [4.53-5.06] |
| 65-74 | -0.09^***^ | 1.73^***^ |
|  | [-0.11 - -0.07] | [1.50-1.96] |
| 75-84 | 0.00 | 0.00 |
|  | -- | -- |
| 85+ | 0.09^***^ | -0.61^***^ |
|  | [0.06 - 0.12] | [-0.90--0.31] |
| Observations | 323630 | 102605 |
| Notes: ^*^ *p* < 0·05, ^**^ *p* < 0·01, ^***^ *p* < 0·001 | | |

**Table S14 – Age-stratified deprivation-related inequalities in HbA1c value at the time of T2DM diagnosis**

|  | 15-34 | 35-44 | 45-54 | 55-64 | 65-74 | 75-84 | 85+ |
| --- | --- | --- | --- | --- | --- | --- | --- |
|  |  |  |  |  |  |  |  |
| **IMD Quintile** |  |  |  |  |  |  |  |
| Q1 (most) | 0.95 | -0.72 | -0.77^*^ | -0.29 | 0.58^*^ | 0.7^*^ | 0.32 |
|  | [-1.21-3.33 | [-1.88-0.50] | [-1.48--0.05] | [-0.86-0.31] | [0.08-1.10] | [0.16-1.26] | [-0.48-1.15] |
| Q2 | 0.79 | -1.01 | -0.17 | -0.02 | 0.54^*^ | 0.16 | 0.81^*^ |
|  | [-1.41-3.22] | [-2.19-0.24] | [-0.91-0.59] | [-0.62-0.60] | [0.03-1.05] | [-0.34-0.67] | [0.04-1.61] |
| Q3 | 0.45 | -0.43 | -0.38 | -0.47 | 0.61^*^ | 0.25 | 0.6 |
|  | [-1.82-2.96] | [-1.68-0.90] | [-1.14-0.40] | [-1.07-0.14] | [0.11-1.13] | [-0.24-0.75] | [-0.15-1.37] |
| Q4 | 0.01 | -0.55 | -0.13 | -0.14 | 0.25 | 0.19 | -0.4 |
|  | [-2.42-2.69] | [-1.91-0.88] | [-0.93-0.70] | [-0.77-0.51] | [-0.25-0.76] | [-0.29-0.69] | [-1.07-0.29] |
| Q5 (least) | 0.00 | 0.00 | 0.00 | 0.00 | 0.00 | 0.00 | 0.00 |
|  | -- | -- | -- | -- | -- | -- | -- |
| Observations | 4915 | 12415 | 23770 | 25495 | 20610 | 11800 | 3595 |
| Notes: ^*^ *p* < 0·05, ^**^ *p* < 0·01, ^***^ *p* < 0·001 | | | | | | | |

**Table S15 – Inequalities in HbA1c value at the time of T2DM diagnosis related to having had NDH previously identified**

|  | Difference in HbA1c (mmol/l) -  T2DM diagnosis |
| --- | --- |
| **Previous NDH** |  |
| Previous diagnosis | 0.00 |
|  | -- |
| No previous diagnosis | 6.52^***^ |
|  | [6.34-6.69] |
|  |  |
| Observations | 102605 |
| Notes: ^*^ *p* < 0·05, ^**^ *p* < 0·01, ^***^ *p* < 0·001 | |

**Table S16 – Age-stratified inequalities in HbA1c value at the time of T2DM diagnosis related to having had NDH previously identified**

|  | 15-34 | 35-44 | 45-54 | 55-64 | 65-74 | 75-84 | 85+ |
| --- | --- | --- | --- | --- | --- | --- | --- |
|  |  |  |  |  |  |  |  |
| **Previous NDH** |  |  |  |  |  |  |  |
| Yes | 0.00 | 0.00 | 0.00 | 0.00 | 0.00 | 0.00 | 0.00 |
|  | -- | -- | -- | -- | -- | -- | -- |
| No | 7.64^***^ | 7.84^***^ | 7.36^***^ | 6.15^***^ | 4.29^***^ | 2.79^***^ | 1.8^***^ |
|  | [6.33-9.025] | [7.17-8.53] | [6.93-7.80] | [5.80-6.50] | [3.99-4.60] | [2.47-3.11] | [1.31-2.30] |
| Observations | 4915 | 12415 | 23770 | 25495 | 20610 | 11800 | 3595 |
| Notes: ^*^ *p* < 0·05, ^**^ *p* < 0·01, ^***^ *p* < 0·001 | | | | | | | |

**Table S17 – Deprivation-related inequalities in HbA1c value at the time of NDH identification or T2DM diagnosis, stratified by whether or not individuals had NDH previously identified**

|  | Previous NDH recorded - Difference in HbA1c (mmol/l) | No previous NDH recorded - Difference in HbA1c (mmol/l) |
| --- | --- | --- |
| **IMD quintile** |  |  |
| Q1 (most deprived) | 0.53^***^ | 1.77^**^ |
|  | [0.22-0.85] | [1.35-2.20] |
| Q2 | 0.59^***^ | 1.32^**^ |
|  | [0.27-0.92] | [0.90-1.75] |
| Q3 | 0.38^***^ | 0.69^*^ |
|  | [0.05-0.71] | [0.27-1.12] |
| Q4 | 0.1 | 0.23 |
|  | [-0.23-0.44] | [-0.20-0.67] |
| Q5 (least deprived) | 0.00 | 0.00 |
|  | -- | -- |
| Observations | 68600 | 34010 |
| Notes: ^*^ *p* < 0·05, ^**^ *p* < 0·01, ^***^ *p* < 0·001 | | |

**Table S18 – Age-stratified deprivation-related inequalities in HbA1c value at the time of NDH identification or T2DM diagnosis, stratified by whether or not individuals had NDH previously identified**

|  | Previous NDH diagnosis - Difference in HbA1c (mmol/l) | No previous NDH diagnosis - Difference in HbA1c (mmol/l) |
| --- | --- | --- |
| **Age group** |  |  |
| 15-34 | 6.54^***^ | 11.4^***^ |
|  | [5.58-7.53] | [10.68-12.14] |
| 35-44 | 4.68^***^ | 9.74^***^ |
|  | [4.20-5.17] | [9.21-10.27] |
| 45-54 | 3.79^***^ | 8.36^***^ |
|  | [3.46-4.11] | [7.92-8.81] |
| 55-64 | 2.39^***^ | 5.75^***^ |
|  | [2.12-2.66] | [5.35-6.16] |
| 65-74 | 0.79^***^ | 2.3^***^ |
|  | [0.56-1.03] | [1.94-2.67] |
| 75-84 | 0.00 | 0.00 |
|  | -- | -- |
| 85+ | -0.07^***^ | -1.05 |
|  | [-0.41-0.27] | [-1.49--0.61] |
| Observations | 68600 | 34010 |
| Notes: ^*^ *p* < 0·05, ^**^ *p* < 0·01, ^***^ *p* < 0·001 | | |

**Table S19 – Age-stratified deprivation-related inequalities in HbA1c value at the time of NDH recording**

|  | 15-34 | 35-44 | 45-54 | 55-64 | 65-74 | 75-84 | 85+ |
| --- | --- | --- | --- | --- | --- | --- | --- |
|  |  |  |  |  |  |  |  |
| **IMD Quintile** |  |  |  |  |  |  |  |
| Q1 (most) | 0.43^***^ | 0.21^***^ | 0.13^***^ | 0.13^***^ | 0.08^***^ | -0.01 | -0.10^*^ |
|  | [0.21 - 0.65] | [0.11 - 0.30] | [0.07 - 0.18] | [0.09 - 0.18] | [0.04 - 0.12] | [-0.06 - 0.05] | [-0.20 - -0.01] |
| Q2 | -0.17 | -0.06 | 0.04 | 0.10^***^ | 0.07^**^ | 0.04 | 0.04 |
|  | [-0.40 - 0.07] | [-0.16 - 0.04] | [-0.01 - 0.10] | [0.05 - 0.14] | [0.02 - 0.11] | [-0.01 - 0.09] | [-0.05 - 0.14] |
| Q3 | -0.07 | 0.02 | 0.03 | 0.09^***^ | 0.05^**^ | 0.06 | 0.04 |
|  | [-0.32 - 0.17] | [-0.09 - 0.13] | [-0.03 - 0.09] | [0.05 - 0.14] | [0.00 - 0.09] | [0.01 - 0.11] | [-0.04 - 0.13] |
| Q4 | 0.16 | 0.03 | 0.07^*^ | 0.12^***^ | 0.05^**^ | 0.06 | -0.02 |
|  | [-0.10 - 0.42] | [-0.09 - 0.15] | [0.00 - 0.13] | [0.07 - 0.17] | [0.02 - 0.09] | [0.01 - 0.10] | [-0.11 - 0.06] |
| Q5 (least) | 0.00 | 0.00 | 0.00 | 0.00 | 0.00 | 0.00 | 0.00 |
|  | -- | -- | -- | -- | -- | -- | -- |
| Observations | 14085 | 31290 | 62064 | 76950 | 76995 | 48060 | 14210 |
| Notes: ^*^ *p* < 0·05, ^**^ *p* < 0·01, ^***^ *p* < 0·001 | | | | | | | |

**References**

1 Grant RL. Converting an odds ratio to a range of plausible relative risks for better communication of research findings. *BMJ* 2014; **348**: f7450.
